# Identification and Evaluation of Alfalfa Volatiles for Monitoring and Management of *Odontothrips loti* and *Frankliniella occidentalis*

**DOI:** 10.3390/insects16121207

**Published:** 2025-11-27

**Authors:** Yingning Luo, Shuhua Wei, Fang Tang, Mark R. McNeill, Xiongbing Tu, Yanqi Liu, Chen Han, Changqing Qu, Xuewei Yin, Liping Ban

**Affiliations:** 1 Engineering Technology Research Center of Anti-Aging Chinese Herbal Medicine of Anhui Province, Fuyang Normal University, Fuyang 236037, China; ynieng_luo@163.com; 2 State Key Laboratory for Biology of Plant Diseases and Insect Pests, Institute of Plant Protection, Chinese Academy of Agricultural Sciences, Beijing 100193, China; 3 College of Grassland Science and Technology, China Agricultural University, Beijing 100193, China; 4 Institute of Plant Protection, Ningxia Academy of Agriculture and Forestry Sciences, Yinchuan 750002, China; 5 College of Grassland Science, Inner Mongolia Agricultural University, Hohhot 010018, China; 6 Bioeconomy Science Institute, Christchurch 8140, New Zealand; 7 Powerchina Huadong Engineering Corporation Limited, Hangzhou 311100, China

**Keywords:** thrips, *Medicago sativa*, semiochemicals, volatile organic compounds, olfactory cues

## Abstract

Plant-derived volatile organic compounds (VOCs) are widely used as insect attractants for population monitoring, offering an efficient and eco-friendly approach to pest management. Since thrips are the dominant pest species in alfalfa (*Medicago sativa*) crops, alfalfa volatiles produced in the presence of *Odontothrips loti* and compounds with attractive potential to both *O*. *loti* and *Frankliniella occidentalis* were investigated. Using gas chromatography–mass spectrometry (GC-MS), 96 VOCs were identified from seven alfalfa cultivars with varying levels of thrips resistance with and without damage by *O*. *loti*. Eleven volatiles were selected for Y-tube olfactometer assays. The semiochemical *p*-Menth-8-en-2-one, which was suppressed in alfalfa subject *O*. *loti* damage, significantly attracted both *O*. *loti* and *F*. *occidentalis* in Y-olfactometer bioassays; the response rates were 2.05~3.07 times compared to control (*p* < 0.05). Further experiments confirmed *p*-Menth-8-en-2-one, dispensed through PE (Polyethylene) vials, was the most effective lure material with the concentrations of 10 ng/μL. This study demonstrated that the alfalfa-derived volatile *p*-Menth-8-en-2-one, shows significant attraction to thrips, which can be utilized for monitoring and management of *odontothrips loti* and *frankliniella occidentalis*.

## Simple Summary

Thrips are a dominant and severe pest in alfalfa, and their rapid development of resistance to chemical insecticides necessitates the development of alternative control strategies. This study investigated alfalfa-derived volatile organic compounds as eco-friendly thrip attractants. We identified that *p*-Menth-8-en-2-one, a volatile in alfalfa that reduced emission after damage by *Odontothrips loti*, could function as a potent attractant for both *O*. *loti* and *Frankliniella occidentalis*. In Y-tube olfactometer assays, thrips response to *p*-Menth-8-en-2-one was 2.05 to 3.07 times greater than control. The optimal application was determined to be a 10 ng/μL concentration released from a polyethylene vial. Overall, our finding establishes *p*-Menth-8-en-2-one as a highly promising semiochemical for early monitoring thrips populations.

## 1. Introduction

For *Medicago sativa* L. (alfalfa), thrips are the main pest species attacking all stages of the plant, which includes a range of taxa from the genera *Apterothrips*, *Frankliniella*, *Haplothrips,* and *Odontothrips* [1,2,3]. *Odontothrips loti* (Haliday, 1852) (Thysanoptera: Thripidae) is an oligophagous insect primarily feeding on legumes, particularly alfalfa. Both nymphs and adult *O*. *loti* can damage the entire alfalfa plant, especially in regions in China where alfalfa is widely cultivated, with infestation rates of over 70% reported [4]. The western flower thrips *Frankliniella occidentalis* (Pergande, 1895) is a polyphagous pest attacking a range of plant species including alfalfa, cucumber, eggplant, and so on [5,6]. Substantial quality and yield losses in alfalfa crops can occur under high thrips densities with an increase in tannin and lignin content and decrease in plant height and leaf area [7,8]. These yield losses are a result of direct feeding on plants and indirectly through the transmission of plant pathogenic viruses (e.g., alfalfa mosaic virus) [9,10]. Research has shown that for every additional thrip counted/6 stems, yield loss increased by 0.026 tonnes/ha (0.0115 tons/acre) [11,12], and in severe infestations, can result in complete loss of the alfalfa stand [13].

Biological traits such as a short life cycle, high fecundity, cryptic behavior, and the haplodiploidy sex determination system in thrips predisposes them to rapid evolution of resistance to insecticides, which significantly increases the challenge of controlling thrips, especially in the context of widespread insecticide use [14]. The financial cost of applying insecticides to control thrips in soybean crops was estimated to be USD 11.4/ha compared to USD 3.2/ha when integrated pest management (IPM) practices were implemented [15]. Therefore, IPM practices like preliminary monitoring and biological control are important to control thrips efficiently, compared to solely using insecticides.

Plants are rich in volatile organic compounds (VOCs) and over 1700 volatile chemicals have been identified and extracted from more than 90 plant families [16]. These volatiles vary with plant development and environmental conditions [17], and play key roles in plant defense against herbivorous insects and pathogens [18], as well as enhancing reproductive success by attracting pollinators and seed dispersers [19]. Additionally, VOCs can provide collective resistance to neighboring plants [20]. Plant volatiles are also important in mediating communication between insects and plants; feeding by insect herbivorous can change the volatiles released by plants, including triggering the synthesis of new compounds known as herbivore-induced plant volatiles (HIPVs) [21]. That makes them promising candidates as repellents or attractants for IPM [22,23]

Push–pull strategies involve the use of behavior-modifying stimuli to manipulate the distribution and abundance of both pest and beneficial insects, thereby reducing pest populations on the crop [24]. with regard to thrips, plant volatiles play an important role in thrips–host interactions. Some volatiles are specific to certain thrips species. For example, cis-jasmone from *Lonicera japonica* (Thunberg) showed significant attraction to New Zealand flower thrips, *Thrips obscuratus* [25], while other volatiles have a broad-spectrum response in multiple species, such as methyl anthranilate, which is attractive to four species of flower thrips, *T*. *hawaiiensis* (Morgan), *T*. *coloratus* Schmutz, *T*. *flavus* Schrank and *Megalurothrips distalis* (Karny), respectively [26]. There are also volatiles that act in the mixture state; a mixture of four compounds composed of dihydromarigold, (Z)-3-hexenyl acetate, limonene, and (Z)-*β*-ocimene can repel *Megalurothrips sjostedti* (Trybom) [27]. In large scale trapping operations, the use of VOCs on sticky boards has been shown to be an effective method to reduce thrips populations [28,29], while having the advantage of being safe for natural enemies, humans, and the environment [30,31]. Currently, semiochemical VOCs derived from plants have proven effective in the monitoring and control of thrips, beetles, lepidopterans, and a wide range of other pests [32,33,34].

Although the research on the interaction between plant volatiles and insects has been very extensive, research on volatile compounds associated with alfalfa and impacts on the behavior of arthropod herbivore pests are relatively unexplored. In this study, we analyzed the composition of volatiles from seven alfalfa cultivars with varying levels of resistance to *O*. *loti* and examined the changes in these volatiles with and without thrips damage. The effect of the semiochemical *p*-Menth-8-en-2 was evaluated through laboratory-based behavioral experiments. Our results contribute to the development of a specific and efficient approach for managing thrips in alfalfa crops to reduce both yield losses and control costs.

## 2. Materials and Methods

### 2.1. Insects and Plants

*Odontothrips loti* and *Frankliniella occidentalis* were initially collected from an alfalfa field at the Shangzhuang Experimental Station of China Agricultural University (40°8′15″ N, 116°11′18″ E) in 2021. The two species were kept separate and subsequently maintained on alfalfa leaves over 3 generations in an artificial climate incubator at 25 ± 1 °C and 60 ± 5% relative humidity, and a light/dark cycle of 16 h:8 h. For laboratory behavioral experiments, we selected healthy adults with similar body size.

Seven alfalfa cultivars used in this study were sourced from the Grassland Protection Laboratory of China Agricultural University. Baralfa 421Q and Xunlu are classified as highly resistant to thrips, Zhongmu No.1 and Gannong No.5 exhibit moderate resistance, while WL343HQ, WL354HQ, and Bara310SC are not resistant [35,36]. Seeds from each cultivar were sown in 25 pots (30 seeds/pot, 1.8 L) and grown in a research greenhouse for two months. A hexahedral cage (45 cm × 55 cm × 40 cm) covered with a 200-mesh nylon over the outside of the potted plant was used to exclude thrips during this time. Plants were raised in the greenhouse, the ambient day and night temperature was 26 °C and 20 °C, respectively, with 60% relative humidity and a light/dark cycle of 16:8 h. After two months, the alfalfa plants reached approximately 30 cm in height, with plants showing similar height and architecture selected for subsequent experiments.

### 2.2. Collection of Volatiles of Alfalfa with and Without O. loti

To investigate the differences in the composition and content of volatiles released by each of the seven alfalfa cultivars in response to thrips feeding, in August (summer) alfalfa volatiles were collected onto 30 mg Super-Q (Alltech Associates Inc., Deerfield, IL, USA) positioned in an adsorbent glass tube by dynamic headspace adsorption.

Alfalfa without or with the addition of *O*. *loti* provided the control and treatment groups, respectively. Potted plants were wrapped in aluminum foil to cover the pots and soil surface; each sampling place three pots in a clean glass gas collection bottle (100 cm height × 20 cm diameter) as one biological replicate. For each cultivar, six independent biological replicates were established for both the control (without thrips) and treatment (with thrips). Before commencing headspace collection, the gas collection bottles were vented for 30 min with the maximum constant flow rate of 400 mL to remove volatile background. During the entire exhaust phase, no adsorbent was added to the glass tube. After the exhaust phase, the absorbent was added to the tubes, and headspace volatiles were collected for eight hours (09:00–17:00 h) for both control and treatments, respectively. For the treatment group, 50 female adults were released onto each plant (150 thrips in total for each replicate) using a fine artists paintbrush (No. 000). They were kept on the plants for 12 h (21:00–09:00 h) before volatile collection commenced; during this period a constant flow rate (400 mL/min) was maintained for collection. Other operations were consistent with that of the control group.

Air was drawn through activated carbon, molecular sieve, silica gel, gas collection bottles, and finally filtered by a glass tube containing the 30 mg Super-Q adsorbent to capture alfalfa volatile. The adsorbent was first added to the glass tube, then plugged with a small amount of glass wool and rinsed with 1 mL dichloromethane. After the dichloromethane was completely volatilized at room temperature, the glass tube was connected to the gas collecting device. The gas collection bottle was sealed with parafilm to prevent air leakage and connected to a pump (QC-1B, Keanlaobao, Beijing, China). Charcoal filtered air was pumped in at 400 mL/min and drawn through an adsorbent glass tube. At the end of the eight hours collection, the adsorbent was eluted with 200 μL of 1 ng/μL nonyl acetate solution, collected into a 2 mL injection bottle containing a liner, and stored at −20 °C until further analysis.

### 2.3. Identification of Alfalfa Headspace Volatiles

The collected samples were analyzed using coupled Gas Chromatography–Mass Spectrometry (GC-MS) (Shimadzu GC-MS QP2010 PLUS, Shimadzu, Kyoto, Japan) equipped with an HP-5MS column (30 m × 0.25 mm × 0.25 μm film thickness; Agilent, Santa Clara, CA, USA). The GC program for identifying alfalfa headspace volatiles ran for 44 min, with an initial temperature of 50 °C for 1 min, followed 230 °C at a ramp up rate of 5 °C/min, then finally raised to 280 °C at a rate of 25 °C/min for a 5 min duration. The inlet temperature was set to 250 °C, and the injection volume was 10 μL. Helium was used as the carrier gas with a flow rate of 1 mL/min. Compounds were identified by comparing their mass spectra with the NIST (https://webbook.nist.gov/chemistry, accessed on 1 December 2020). Additionally, the same amount of synthetic standards (acetic acid nonyl ester) were analyzed under the same conditions, and identification was supported by comparing retention times and mass spectra. Principal component analysis (PCA) of volatiles was completed in RStudio 4.2.1 to visually show the difference of volatile compounds in different cultivars.

### 2.4. Olfactometer Assays with Alfalfa Volatiles

Based on the relative content of alfalfa volatiles, we selected eight compounds that exhibited significant differences with and without *O*. *loti* infestation (linalool, 4-pentenal, D-limonene, *p*-Menth-8-en-2-one, Table S1) or had high relative content across both control and thrips treatment groups (nonanal, 2-ethyl-1-hexanol, m-xylene, butylated hydroxytoluene, Table S1), Additionally, three compounds (α-pinene, salicylaldehyde, tridecane) previously reported to attract thrips [37,38,39]. These 11 compounds were used to conduct Y-tube olfactometer bioassays (Table S5).

To assess the behavioral responses of female and male *O*. *loti* to these compounds, the 11 synthetic compounds were dissolved in laboratory-grade paraffin oil (Analytical Reagent Paraffin Liquid, SCRC, Shanghai, China), to a concentration of 10 ng/μL as the test odor source. Paraffin oil only served as the control. The Y-tube olfactometer used in the experiment was constructed from glass and consisted of a central stem and two 10 cm long × 1 cm diameter arms, positioned at an angle of 75°. Air was first purified through activated charcoal before passing through a gas-washing bottle filled with distilled water to mediate the humidity. It was then split into two streams. Each stream flowed through a 100 mL glass flask (containing either the test or control odor source), and was directed into each arm at a flow rate of 50 mL/min. The experiments were conducted in a dark room maintained at a temperature of 26 ± 1 °C, where the olfactometer was illuminated by a 28 W LED light positioned at the head of the Y-tube. Teflon tubing was used to connect the components of the system.

Newly emerged female and male thrips were collected and placed in separate plastic vented containers (8.5 cm dia. × 6.5 cm high) containing excised alfalfa (cv. Zhongmu No.1) leaves grown in the glasshouse. To reduce desiccation, two lentils from plants grown in the glasshouse were also added to the containers. The containers were maintained in an insect rearing room at 26 °C and 60% relative humidity, and a light/dark cycle of 16 h:8 h. Thrips were 3–5 days old when the bioassays commenced and starved for three hours prior to undertaking the bioassay.

For each bioassay, a single thrips was introduced into the stem of the Y-tube, and its behavior was observed for five minutes. The choice was recorded when the thrips entered halfway into one arm of the Y-tube and remained there for at least one minute. If the thrips did not enter either arm during the observation period, it was recorded as “no choice.” After consecutively testing five thrips, the positions of the odor sources were swapped to eliminate any directional bias in the apparatus. After testing ten thrips, the Y-tube was disconnected and cleaned with 75% ethanol and dried in an oven at 60 °C for 30 min. Each 60 biological replicates for female and male were carried out for each treatment. Thrips were handled using an artist’s paintbrush (No. 000).

### 2.5. Effect of p-Menth-8-en-2-one Concentration on the Behavioral and Electrophysiological Responses of Thrips

#### 2.5.1. Behavioral Responses

After clarifying the attraction of *p*-Menth-8-en-2-one to *O*. *loti*, further behavioral assays were constructed to test the response of both *O*. *loti* and *F*. *occidentalis* to *p*-Menth-8-en-2-one under different concentrations. We tested the response of female *O*. *loti* to six concentrations (1, 5, 10, 50, 100 and 1000 ng/μL *p*-Menth-8-en-2-one). Because of limited numbers, male *O*. *loti* were tested at only 10, 100, and 1000 ng/μL *p*-Menth-8-en-2-one. The response of both male and female *F*. *occidentalis* were also tested at 10, 100, and 1000 ng/μL *p*-Menth-8-en-2-one. All bioassays were conducted using the Y-tube olfactometer following the methodology described above.

#### 2.5.2. Effect of Lure Type and *p*-Menth-8-en-2-one on the Behavioral Response of *O*. *loti*

To assess the responses of female *O*. *loti* to different lure types as sustained-release carriers for *p*-Menth-8-en-2-one, three lures were evaluated: rubber plug (green hollow rubber, 20 mm × 9 mm × 6 mm, Pherobio, Beijing, China), PVC pipe (white, 180 mm length × 5 mm diameter, Pherobio, Beijing, China), and PE vial (1 mL volume, Pherobio, Beijing, China). These lures were immersed in one of four concentrations of *p*-Menth-8-en-2-one (10, 100, 1000, and 10,000 ng/μL), or paraffin oil (control) for 24 h. The lures were then removed and dried for 2 h at 26 °C in the laboratory, after which they were placed in 100 mL glass flask bottles. No replacement of lures was necessary during the bioassay. The Y-tube olfactometer bioassay was conducted as described above. Assays where no choice was made were excluded from subsequent analysis.

#### 2.5.3. Electroantennography Assays

To detect the electrophysiological responses of both female *O*. *loti* and *F*. *occidentalis* to synthetic *p*-Menth-8-en-2-one, the insect antennal potential measurement system (IDAC2, Syntech, Kirchzarten, Germany) was utilized. Glass electrodes were prepared using a P-97 Flaming/Brown™ micropipette puller (Sutter, Novato, CA, USA), and a 0.1 mol/L KCl solution was used as the electrolyte. *p*-Menth-8-en-2-one was first dissolved in paraffin oil and then serially diluted to concentrations of 1, 5, 10, 100, and 1000 ng/μL for *O*. *loti*, and 1, 10, 100, 500, and 1000 ng/μL for *F*. *occidentalis*, respectively. Either 10 μL of liquid paraffin oil (control) or *p*-Menth-8-en-2-one was added to the filter paper strip (0.5 cm × 10 cm) then inserted into a glass Pasteur pipette (150 mm, Fisher Scientific, Waltham, MA, USA) for testing [22].

Under a microscope, the top one third of the head of a female adult thrip (3–5 days old) was separated from the body with a sterile scalpel and one of the antennae removed. The base of the head segment was placed onto the tip of the indifferent electrode, and the tips of the antennae were positioned into the end of the recording electrode. Antennae were tested in order from low to high concentration, and the strength of the response measured. The paraffin oil control was tested at the beginning and end of each bioassay and the mean value taken. Simultaneous electroantennogram signals were obtained and recorded with specialized software (GC-EAD 2.3; Syntech, Hilversum, The Netherlands). Each treatment included 8 biological replicates for *O*. *loti*, and 10 for *F*. *occidentalis*, respectively. The time between treatments was generally 30–60 s.

#### 2.5.4. Statistical Analysis

Internal standard method was used to quantify the volatiles, and the internal standard was nonyl acetate (CAS:143-13-5). The peak area of the total ion current diagram is taken as the input data for calculation; the formula is as follows:

$$\mathrm{Relative}\;\mathrm{content}\;=\;\frac{\mathrm{Content}\;\mathrm{of}\;\mathrm{volatiles}}{\mathrm{Content}\;\mathrm{of}\;\mathrm{internal}\;\mathrm{standard}}\;=\frac{\mathrm{Peak}\;\mathrm{area}\;\mathrm{of}\;\mathrm{the}\;\mathrm{volatiles}}{\mathrm{Peak}\;\mathrm{area}\;\mathrm{of}\;\mathrm{internal}\;\mathrm{standard}}$$

The multiple unpaired t-test was used to analyze the difference of relative content of volatiles between control and treatment of each cultivar by GraphPad Prism 10.1.1 (GraphPad Software, Boston, MA, USA); One-way ANOVA was used for electrophysiological responses by SPSS 22.0 (IBM, Armonk, NY, USA). The chi-squared test was used to analyze the behavior choice by GraphPad Prism 10.1.1. Principal component analysis was performed in RStudio software (R 4.2.1, https://cran.r-project.org).

## 3. Results

### 3.1. Composition and Relative Content of Volatiles of Alfalfa with and Without O. loti

**Figure 1:**
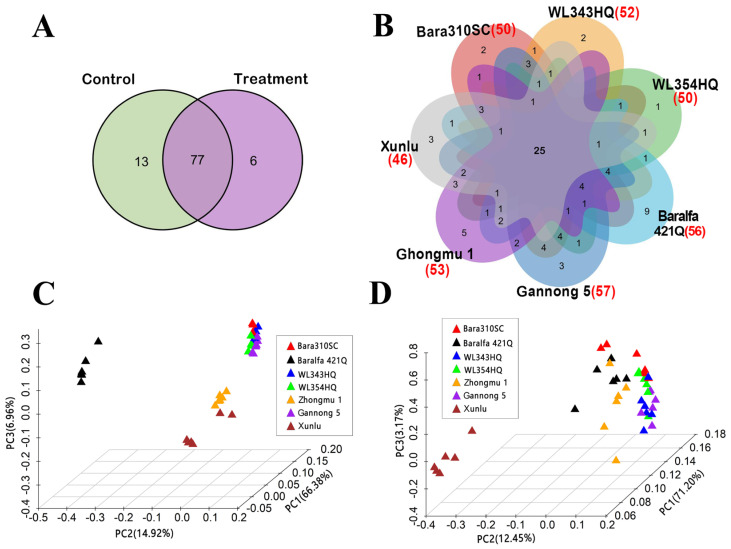
Composition of volatile compounds of control and *Odontothrips loti*-infested plants in all seven alfalfa cultivars (Bara310SC, WL343HQ, WL354HQ, Gannong No.5, Baralfa 421Q, Zhongmu No.1, and Xunlu). (**A**) Venn diagram of the diversity of compounds control (plants without infestation) and treatment (infested plants). (**B**) Venn diagram of the compound diversity for all seven alfalfa cultivars. Numbers in brackets refer to the number of compounds identified for each cultivar. (**C**) Principal component analysis (PCA) of volatiles in the control plants. (**D**) PCA of volatiles in *O*. *loti*-infested plants. Number of replicates for control and thrips-infested plants for each cultivar was six.

Based on GC-MS analysis, the composition and relative content of volatiles of the seven alfalfa cultivars (Baralfa 421Q, Xunlu, Zhongmu No.1, Gannong No.5, WL343HQ, WL354HQ, and Bara310SC) were different with and without damage by *O*. *loti*. A total of 96 volatiles (Table S1) were collected, comprising 14 alcohols, 13 aldehydes, 6 ketones, 4 terpenes, 3 acids, 7 esters, 19 hydrocarbons, 17 aromatic hydrocarbons, 2 phenols, and 11 unidentifiable compounds. In total, 90 compounds were identified in undamaged alfalfa (control), while 83 were associated with thrips-damaged alfalfa (Figure 1A). Among these, 13 compounds only appeared in the control, and most of them came from cv. Baralfa 421Q (Table S2). Six compounds were only detected in the thrips exposed group, the majority of which were from cv. Xunlu (Table S3).

We compared volatiles amongst the cultivars and found that an average of 50 volatiles were detected for each alfalfa cultivar, of which 25 were common compounds in all seven cultivars (Figure 1B, Table S1, indicated in red). Principal component analysis of volatiles produced by the seven alfalfa cultivars that had not been exposed to thrips found that the first three principal components (PC1: 66.38%; PC2: 14.92%; PC3: 6.96%) explained 88.26% of the variables (Figure 1C). Both cv. Baralfa 421Q and cv. Xunlu (both highly resistant to thrips) and cv. Zhongmu No.1 (moderate resistance) could be clearly distinguished from the remaining cultivars. For the seven cultivars exposed to *O*. *loti*, the first three principal components (PC1: 71.20%; PC2: 12.45%; PC3: 3.17%) explained 86.82% of the variables, while cv. Xunlu could clearly be distinguished from the other cultivars (Figure 1D). The PCA results showed that thrip infection eliminated the specificity of the volatile composition of the highly resistant cv. Baralfa 421Q. Therefore, we speculated that the significantly changed volatile components of this cultivar without and with *O*. *loti* infestation might be involved in its resistance to thrips.

**Figure 2:**
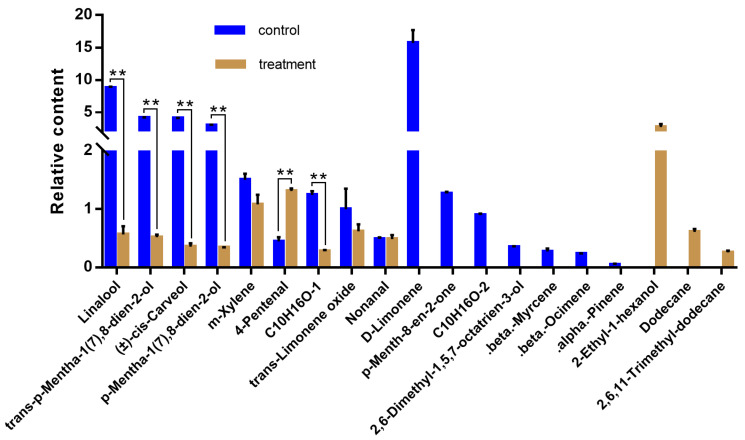
Mean (±SEM) changes in the relative content of identified volatile compounds without and with *Odontothrips loti* infestation in Baralfa 421Q. Multiple unpaired t-test was used to analyze the difference of relative content of each volatile between control and treatment; the significances of the changes are ** *p* < 0.01. Unknown compounds are represented by molecular formulae. Number of replicates was six.

Among the 25 compounds commonly shared by all seven alfalfa cultivars, we found 2-ethyl-1-hexanol, nonanal, m-xylene, butylated hydroxytoluene, and hexadecane had a high relative content compared to the other compounds (Table S4). Differences were observed among cultivars. For instance, cv. WL343HQ and cv. WL354HQ had the highest relative content of butyltoluene, whereas cv. Baralfa 421Q and cv. Xunlu had the highest relative content of linalool. Additionally, unique compounds in alfalfa appeared or disappeared following *O*. *loti* damage across the seven cultivars, indicating inter-cultivar variation (Figure S1), although their relative contents were low. Notably, in cv. Baralfa 421Q, the relative content of D-limonene and *p*-Menth-8-en-2-one was high prior to *O*. *loti* infestation, but these compounds were absent following thrips infestation. This suggested that thrips damage had a certain effect on the release of host volatiles (Figure 2, Tables S1 and S2).

### 3.2. Behavioral and Electroantennographic Responses of Thrips to Alfalfa Volatiles

**Figure 3:**
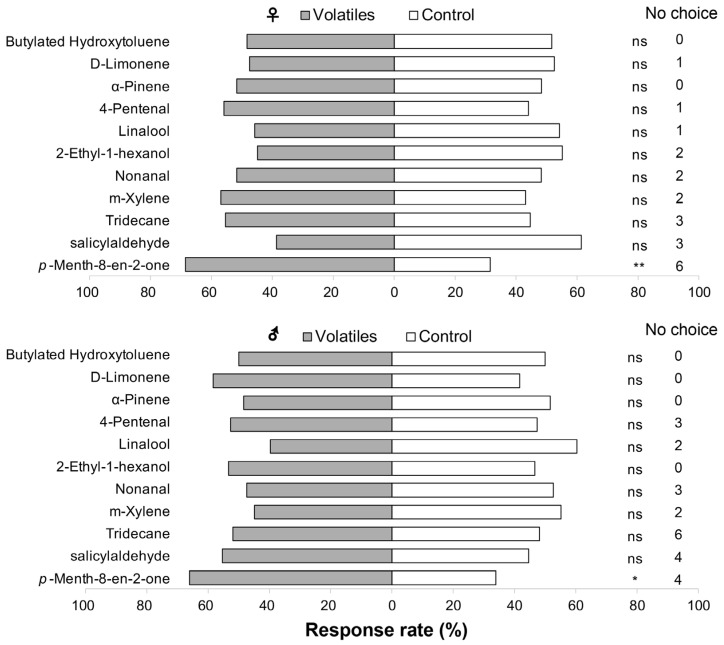
Responses of adult female (♀) and male (♂) *Odontothrips loti* to 11 volatiles in a Y-tube olfactometer. Chi-squared test, df = 1, the significances in response indicated as follows: ns—no significant difference, * *p* < 0.05, and ** *p* < 0.01. Total number of replicates for male and female for each compound was sixty. The number of thrips not responding was between 0 to 10%.

Eleven volatiles with high relative content or significant changes in content without and with *O*. *loti* damage were selected for Y-tube olfactometer assays. The assay results showed that *p*-Menth-8-en-2-one significantly attracted both male (χ^2^ = 5.786, df = 1, *p* = 0.016) and female (χ^2^ = 7.407, df = 1, *p* = 0.006) *O*. *loti* compared to the control (Figure 3, Table S6). The other compounds elicited no significant responses by both male and female thrips.

**Figure 4:**
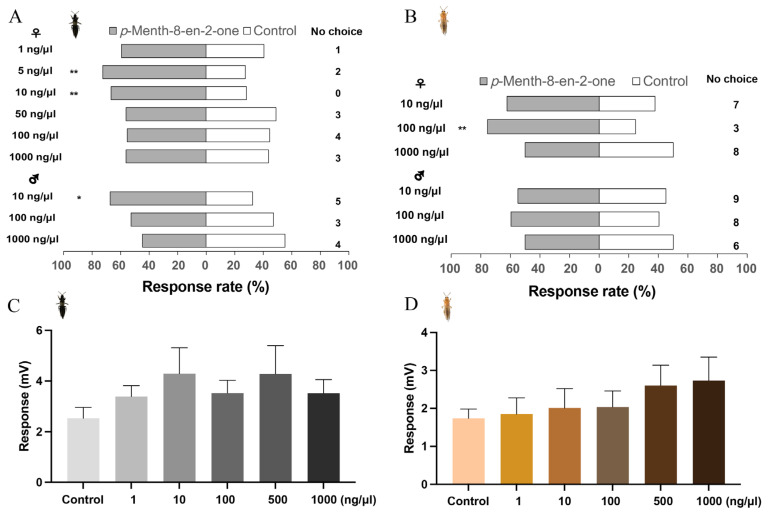
Effect of different concentrations of *p*-Menth-8-en-2-one on the behavioral (%) and EAG (millivolt, mV) responses by adult female *Odontothrips loti* and *Frankliniella*. *occidentalis*, respectively. *Odontothrips loti* behavioral (**A**) and EAG (**C**) responses to *p*-Menth-8-en-2-one in relation to the control compound (paraffin oil); number of replicates for each concentrate was eight. *Frankliniella occidentalis* behavioral (**B**) and EAG (**D**) responses to *p*-Menth-8-en-2-one in relation to the control; number of replicates for each concentrate was ten. Chi-squared test was used for behavioral data, df = 1, the significances in response to indicated as * *p* < 0.05 and ** *p* < 0.01, total number of replicates for male and female for each concentrate was sixty, the number of *O*. *loti* and *F*. *occidentalis* not responding was between 0 to 8% and 5–15%, respectively. One-way ANOVA was performed for EAG data, no significances detected.

**Figure 5:**
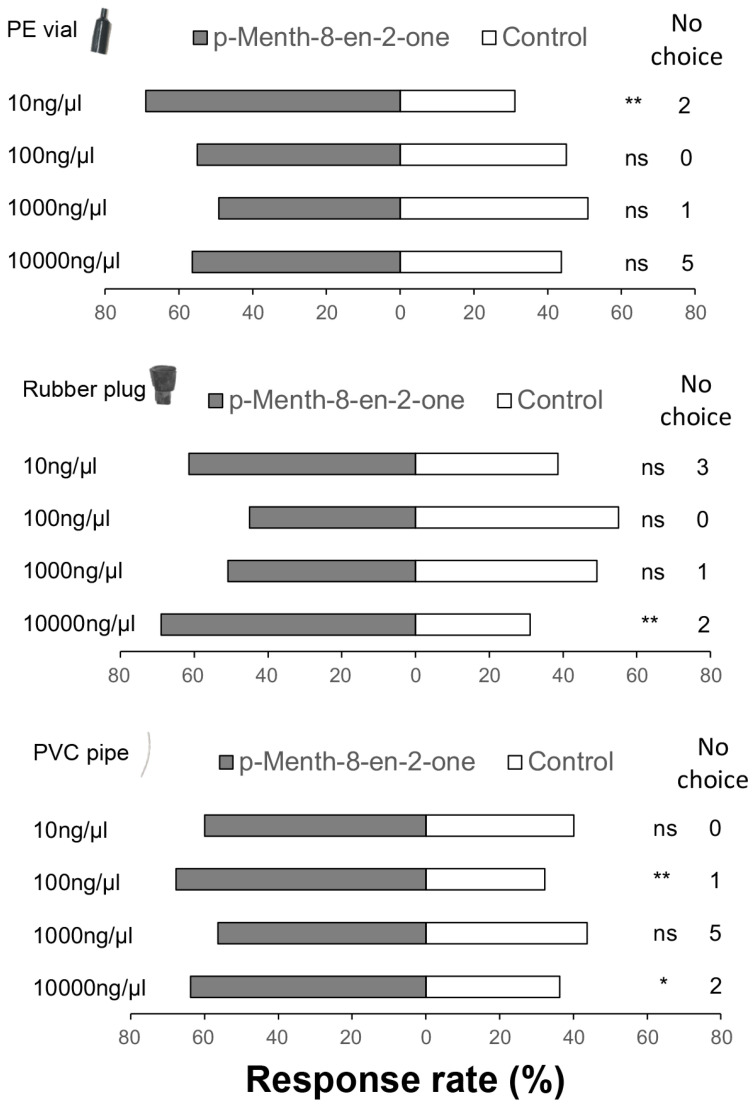
Influence of PE vial, rubber plug, and PVC-piped lures on the behavioral response of adult female *Odontothrips loti* to four different concentrations (10–10,000 ng/µL), in relation to the control compound (paraffin oil). Chi-squared test, df = 1, the significance in response to *p*-Menth-8-en-2-one and control (paraffin oil) carried by three different lures indicated as * *p* < 0.05 and ** *p* < 0.01, ns means no significant difference. Total number of replicates for female *O*. *loti* for each concentration was sixty. The number of *O*. *loti* not responding was between 0 to 8%.

To determine the application range and dosage of *p*-Menth-8-en-2-one as a potential thrips attractant, Y-tube behavior experiments were conducted on adult *O*. *loti* and *F*. *occidentalis* at various concentrations. Overall, there was a very low percentage of thrips that failed to make a choice ranging from 0 to 15% (n = 9) (Figure 3, Figure 4 and Figure 5). We found that *p*-Menth-8-en-2-one was the most attractive to both male (χ^2^ = 6.564, df = 1, *p* = 0.01) and female (χ^2^ = 9.281, df = 1, *p* = 0.002) adults of *O*. *loti* at 10 ng/μL, with females showing a stronger attraction than males at this concentration, 2.35 times response rate compare to control (*p* = 0.002 < 0.05) for females, 2.06 times response rate compare to control (*p* = 0.001 < 0.05) for males (Figure 4A). For *F*. *occidentalis*, only the female exhibited a significant attraction of 100 ng/μL (χ^2^ = 14.751, df = 1, *p* = 0.000), 3.07 times response rate compared to control (*p* = 0.002 < 0.05). At 1000 ng/μL, neither male nor female thrips showed any attraction to the compound (*p* = 1.000 > 0.05). Overall, female thrips are more attracted to *p*-Menth-8-en-2-one than male thrips.

We conducted electrophysiological tests on the response of adult females of both *O*. *loti* and *F*. *occidentalis* to *p*-Menth-8-en-2-one. The EAG response of *O*. *loti* females to *p*-Menth-8-en-2-one showed an initial increase followed by a decrease, with the strongest reaction observed at a concentration of 5 ng/μL (Figure 4C). In contrast, the EAG response of *F*. *occidentalis* females to *p*-Menth-8-en-2-one increased steadily as the dose increased (Figure 4D). No significant differences were observed within the specified concentration range for either *O*. *loti* (df = 5, *p* = 0.598) or *F*. *occidentalis* (df = 5, *p* = 0.557).

To identify the most effective lures, we first determined the degree of attraction in a laboratory of three different lure materials (rubber plug, PVC pipe, and PE vial) at concentrations of 10 ng/μL, 100 ng/μL, 1000 ng/μL, and 10,000 ng/μL. The results showed that at a concentration of 10 ng/μL, the PE vial was highly attractive to *O*. *loti* (χ^2^ = 8.345, df = 1, *p* = 0.004), while the rubber plug was significantly attractive to *O*. *loti* when dosed with 10,000 ng/μL *p*-Menth-8-en-2-one(χ^2^ = 8.345, df = 1, *p* = 0.004). The PVC pipe demonstrated a significant attraction effect at the 100 ng/μL (χ^2^ = 7.475, df = 1, *p* = 0.006) and 10,000 ng/μL (χ^2^ = 4.414, df = 1, *p* = 0.036) (Figure 5).

## 4. Discussion

We collected and identified 96 compounds from seven alfalfa cultivars with varying levels of thrips resistance and found that the VOCs collected from the different cultivars exhibited notable differences. Compounds like m-Xylene, p-Xylene, decanal, Tridecane, and nonanal exist in all seven alfalfa cultivars; α-pinene, β-myrcene, and D-Limonene only exist in cultivar Baralfa 421Q without thrip infestation; salicylaldehyde only exists in cultivar Zhongmu No.1 without thrip infestation. Decanal, α-pinene, and β-myrcene are commonly reported in leaf volatile of alfalfa [40,41]. D-limonene is the main component of citrus essential oil [42,43], m-xylene is commonly used as a pesticide a synergist [44], and salicylaldehyde and nonanal are known for their strong nematocidal and bactericidal activity [45], with salicylaldehyde also exhibiting acaricidal activity [46]. Additionally, 2-ethyl-1-hexanol and nonanal play a key role in inhibiting sclerotia activity, limiting the production of ascospores, and reducing disease level [47]. Tridecane has a repellent effect on *Liothrips jatrophae* [37]. These plant secondary metabolites that act as insecticidal or repellent active components are crucial in enhancing plant resistance to pests [48] and are suggested to have potential for application in biological control [49].

Insect infestation has a significant effect on the release of host volatiles, and changes in VOCs may play a role in resisting host invasion. In the most thrips-resistant cultivar, Baralfa 421Q, D-limonene and 2-ethyl-1-hexanol exhibited the highest relative content without and with thrips damage, respectively (Figure 2). Similar results were observed in *Brassica oleracea* var Botrytis, where the production of limonene was significantly reduced by the infestation of *Bagrada hilaris* (Burmeister, 1835) and *Nezara viridula* (Linnaeus, 1758), while an increase in the emission of 2-ethyl-1-hexanol was noted [50]. Both D-limonene and 2-ethyl-1-hexanol elicit behavioral responses in various insect species, but these responses appear to be dose-dependent and species-specific, showing attraction, repellency, or even insect toxicity under different conditions [43,51,52,53]. Furthermore, numerous studies have highlighted the dynamic role of herbivore-damaged plants in attracting natural enemies [54]. For instance, French marigold, which contains abundant volatile compounds such as D-limonene and 2-ethyl-1-hexanol, can attract natural enemies of aphids, such as *Harmonia axyridis* (Pallas), while simultaneously repelling the pest *Aphis citricola* [55].

*p*-Menth-8-en-2-one shows significant attractant activity to both O. loti and *F*. *occidentali*. This compound is primarily found in mint [56,57]. Our study is the first report of *p*-Menth-8-en-2-one being detected in alfalfa, also confirming its attraction to two thrips species. Behavioral experiments indicate that both female and male *O*. *loti* are more sensitive to *p*-Menth-8-en-2-one than *F*. *occidentalis*, and female thrips are more attracted to *p*-Menth-8-en-2-one than male thrips (Figure 4), indicating that the interaction with the same VOC differs between species, as well as sex. Our previous works have demonstrated that *p*-Menth-8-en-2-one has a strong binding affinity for O. lotOBP6, an odorant-binding protein specifically expressed in the antennae [4], suggesting that *p*-Menth-8-en-2-one plays a species-specific role in host plant searching and localization by *O*. *loti*. Following thrips damage, *p*-Menth-8-en-2-one was depleted in cv. 421Q (highly resistant to thrips), and its levels exhibited a synergistic trend with D-limonene, likely since D-limonene serves as a precursor in the synthesis of *p*-Menth-8-en-2-one [58]. In this study, we hypothesize that alfalfa may reduce its attraction to thrips by decreasing *p*-Menth-8-en-2-one levels, while potentially increasing its attraction to predators such as *H*. *axyridis* by elevating 2-ethyl-1-hexanol levels. This hypothesis requires further experimental validation.

The innovative use of attractants or repellents has been demonstrated as a promising pest management tool for future farming systems. However, challenges such as high volatility and instability of these compounds need to be addressed for their effective application in push–pull farming system [59]. In this study, we tested the attraction of three lure materials at four concentrations; the PE vial showed significant attraction at lowest concentration (10 ng/μL), followed by PVC pipe (100 ng/μL and 10,000 ng/μL), then the rubber plug (10,000 ng/μL). Therefore, from an efficacy point of view, the use of PE vials or PVC pipe are better than rubber plugs. We speculate that this is because the rubber plug has a more impervious surface, and lacks a hollow structure compared to the two other lures, and therefore less of the semiochemical is able to be loaded onto it. These characteristics contribute to a faster volatilization of *p*-Menth-8-en-2-one from the rubber plug, making it less effective as a lure to monitor thrips under field conditions. Loading the rubber plug with a higher dose could potentially achieve a longer duration of activity, but a higher concentration may mean *p*-Menth-8-en-2-one acts as a repellent, at least initially, until concentration drops to a level where it is attractive. But further experiments are needed to confirm this view.

For field application, insect attractants are often used for early detection and prediction of insect situation, and are usually used in conjunction with sticky boards, which has demonstrated long-lasting and effective trapping results for *Apolygus lucorum* (Meyer-Dur) [60] and *Protaetia brevitarsis* Lewis [61]. A preliminary field trial has been constructed to identify *p*-Menth-8-en-2-one as a suitable attractant trap design that could be employed with sticky board to monitor *O*. *loti* and *F*. *occidentalis* (Luo et al., unpublished data [62]). Developing a working system of monitoring thrips to provide early warning requires timing of the deployment of traps, the spacing of traps, their position in the field (margins or center of crop, upwind or downwind), trap height, lure type, attractant concentration, and the thresholds (e.g., number of thrips/traps) for implementing control management [63,64,65]. If a robust association can be established between the number of thrips captured on sticky boards and number of thrips occurring in alfalfa plants during the season, this may provide a more effective tool for field managers to monitor thrips populations and integrated pest management interventions when required and before significant yield losses occur.

## 5. Conclusions

The plant volatile *p*-Menth-8-en-2-one has been identified in thrips-damaged alfalfa, and this compound significantly attracts both *O*. *loti* and *F*. *occidentalis*. A series of behavioral and EAG experiments determined the concentration and lure material for thrip application.

## Data Availability

The original contributions presented in this study are included in the article/Supplementary Material. Further inquiries can be directed to the corresponding authors.

## Supplementary Materials

The following supporting information can be downloaded at: https://www.mdpi.com/article/10.3390/insects16121207/s1, Table S1: Relative total content of 96 leaf volatiles identified from the control and *O*. *loti* damaged plants for each of the seven alfalfa cultivars; Table S2: Thirteen volatiles identified in alfalfa cultivars not exposed to *O*. *loti*; Table S3: Six volatiles identified in alfalfa cultivars subject to feeding damage by *O*. *loti;* Table S4: Relative content of 25 compounds common to the seven alfalfa cultivars; Table S5: Synthetic compounds used in olfactometer assays to test attraction by *Odontothrips loti* adults; Table S6: Behavioral response of female and male *O*. *loti* to 11 volatile compounds; Figure S1: Venn diagram and compounds identified from each control and *Odontothrips loti* infested alfalfa cultivar; Figure S2: The ion current diagram for the control treatment of the alfalfa cultivar Baralfa 421Q.
