# Supplementary material for: Identification and Evaluation of Alfalfa Volatiles for Monitoring and Management of Odontothrips loti and Frankliniella occidentalis
Source: Insects. 2025 Nov 27;16(12):1207. doi: 10.3390/insects16121207 (PMC12733796; doi:10.3390/insects16121207)
Supplement: Supplementary file 1 [file insects-16-01207-s001.zip › Supplementary_Materials_Figures.pdf]

Supplement Figure

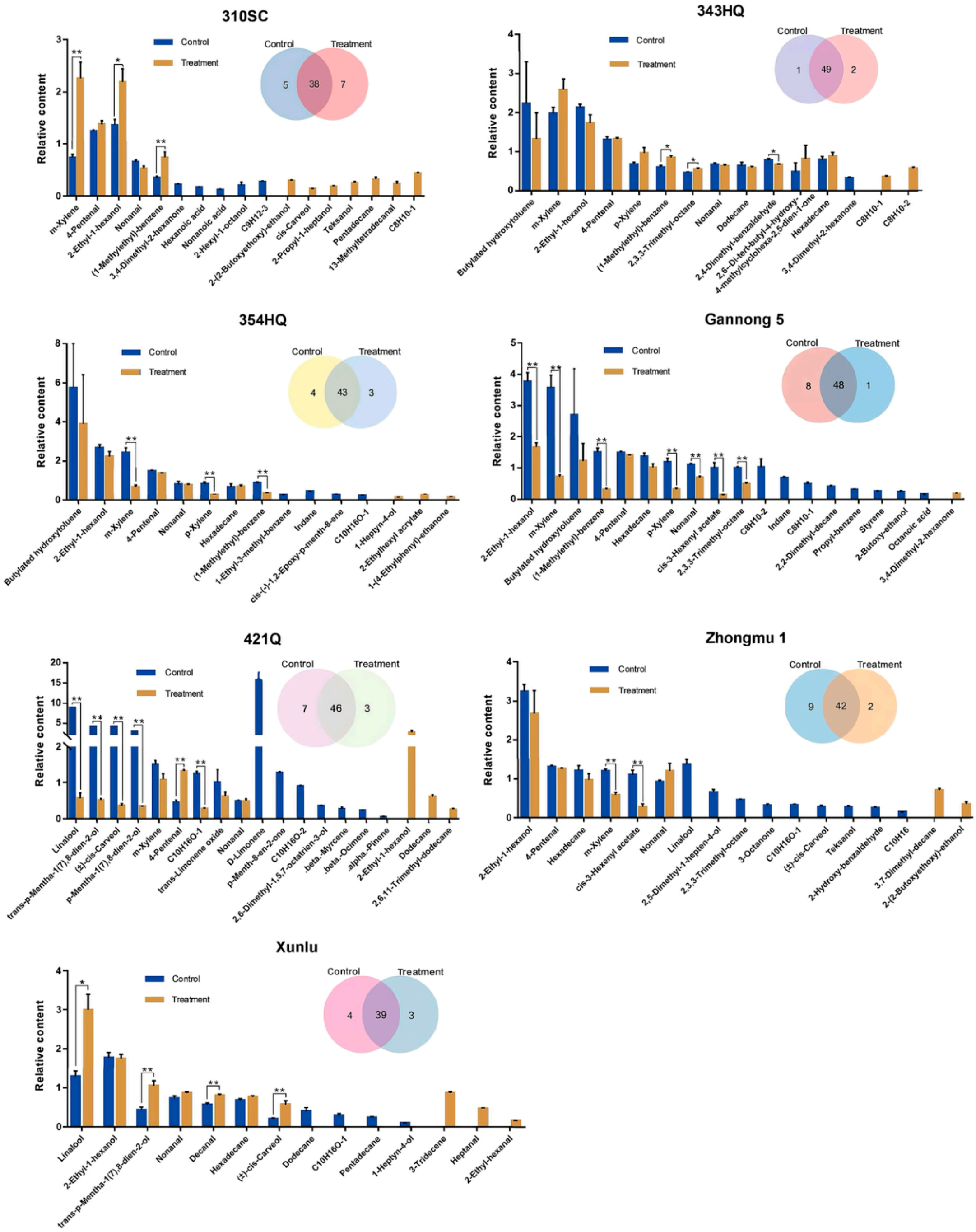

Figure S1. Venn diagram and compounds identified from each control and *Odontothrips loti* infested alfalfa cultivar. Multiple unpaired t-test was used to analyze the difference of relative content of each volatile between control and treatment, the significances in response between the seven control and respective infested cultivars indicated as * $p<0.05$ and ** $p<0.01$. Number of replicates for control and thrips infested plants for each cultivar was six.

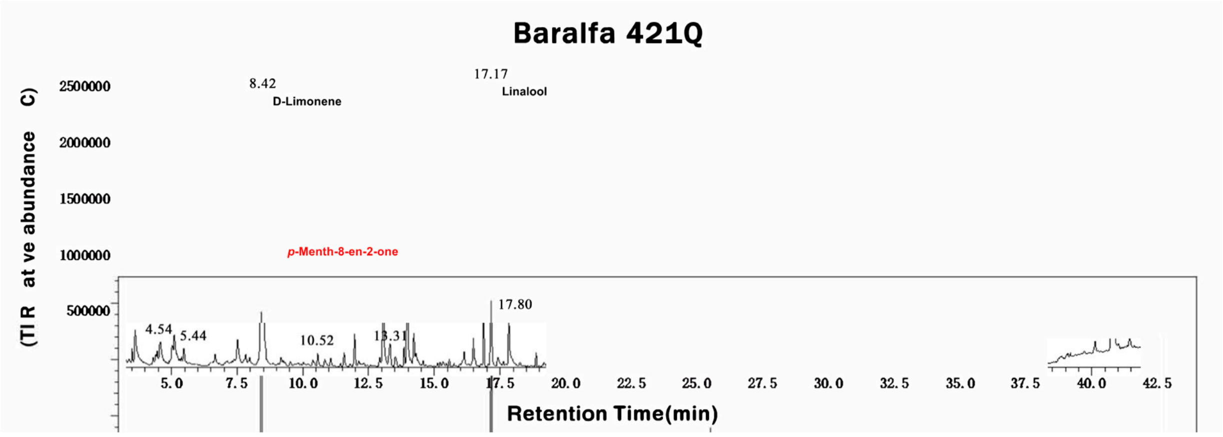

Figure S2. The ion current diagram for the control treatment of the alfalfa cultivar Baralfa 421Q.
